# Reducing Auditory Nerve Excitability by Acute Antagonism of Ca^2+^-Permeable AMPA Receptors

**DOI:** 10.3389/fnsyn.2021.680621

**Published:** 2021-07-05

**Authors:** Amit Walia, Choongheon Lee, Jared Hartsock, Shawn S. Goodman, Roland Dolle, Alec N. Salt, Jeffery T. Lichtenhan, Mark A. Rutherford

**Affiliations:** ^1^Department of Otolaryngology, Washington University in St. Louis School of Medicine, St. Louis, MO, United States; ^2^Department of Communication Sciences and Disorders, University of Iowa, Iowa City, IA, United States; ^3^Department of Biochemistry and Molecular Biophysics, Washington University Center for Drug Discovery, Washington University in St. Louis School of Medicine, St. Louis, MO, United States

**Keywords:** Ca^2+^-permeable AMPA receptor, auditory nerve fiber, cochlear synapse, intracochlear drug, blood labyrinth barrier, hearing protection, non-competitive open-channel block, IEM-1460 and IEM-1925

## Abstract

Hearing depends on glutamatergic synaptic transmission mediated by α-amino-3-hydroxy-5-methyl-4-isoxazolepropionic acid receptors (AMPARs). AMPARs are tetramers, where inclusion of the GluA2 subunit reduces overall channel conductance and Ca^2+^ permeability. Cochlear afferent synapses between inner hair cells (IHCs) and auditory nerve fibers (ANFs) contain the AMPAR subunits GluA2, 3, and 4. However, the tetrameric complement of cochlear AMPAR subunits is not known. It was recently shown in mice that chronic intracochlear delivery of IEM-1460, an antagonist selective for GluA2-lacking AMPARs [also known as Ca^2+^-permeable AMPARs (CP-AMPARs)], before, during, and after acoustic overexposure prevented both the trauma to ANF synapses and the ensuing reduction of cochlear nerve activity in response to sound. Surprisingly, baseline measurements of cochlear function before exposure were unaffected by chronic intracochlear delivery of IEM-1460. This suggested that cochlear afferent synapses contain GluA2-lacking CP-AMPARs alongside GluA2-containing Ca^2+^-impermeable AMPA receptors (CI-AMPARs), and that the former can be antagonized for protection while the latter remain conductive. Here, we investigated hearing function in the guinea pig during acute local or systemic delivery of CP-AMPAR antagonists. Acute intracochlear delivery of IEM-1460 or systemic delivery of IEM-1460 or IEM-1925 reduced the amplitude of the ANF compound action potential (CAP) significantly, for all tone levels and frequencies, by > 50% without affecting CAP thresholds or distortion product otoacoustic emissions (DPOAE). Following systemic dosing, IEM-1460 levels in cochlear perilymph were ~ 30% of blood levels, on average, consistent with pharmacokinetic properties predicting permeation of the compounds into the brain and ear. Both compounds were metabolically stable with half-lives >5 h *in vitro*, and elimination half-lives *in vivo* of 118 min (IEM-1460) and 68 min (IEM-1925). Heart rate monitoring and off-target binding assays suggest an enhanced safety profile for IEM-1925 over IEM-1460. Compound potency on CAP reduction (IC_50_ ~ 73 μM IEM-1460) was consistent with a mixture of GluA2-lacking and GluA2-containing AMPARs. These data strongly imply that cochlear afferent synapses of the guinea pig contain GluA2-lacking CP-AMPARs. We propose these CP-AMPARs may be acutely antagonized with systemic dosing, to protect from glutamate excitotoxicity, while transmission at GluA2-containing AMPARs persists to mediate hearing during the protection.

## Introduction

**Figure 1:**
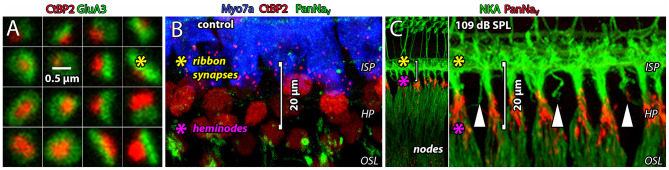
Glutamatergic ribbon synapses and afferent fibers are vulnerable to noise exposure. **(A)** Square tiles (~1 μm^2^) show 16 individual afferent ribbon synapses from the 2nd cochlear turn of a control cochlea. The presynaptic ribbon (red) is a cytoplasmic density anchored to the presynaptic membrane of the IHC. The main synaptic ribbon protein, Ribeye, is labeled with an antibody to CtBP2 which is the B-domain of Ribeye, and itself a nuclear transcription factor (Schmitz et al., 2000). The ribbon is juxtaposed to a large postsynaptic array of AMPARs (green). **(B)** The glutamatergic ribbon synapses in the ISP (red) are ~20 μm from the spike-generating heminodes at the HP (green). **(C)** Immediately after a 90-min exposure to 4–8 kHz octave band noise at 109 dB SPL. Heminodes (red) are positioned at the habenula perforata (HP), just before ANFs become myelinated as they enter the osseous spiral lamina (OSL) toward the ganglion. Some dendrites in the ISP appear to be severed from their heminodes at the HP (vertical arrowheads). Spike-generating heminodes and nodes on ANFs express voltage-gated Na^+^ channels (anti-Pan-NaV, red). In all panels, yellow and magenta asterisks indicate positions of ribbon synapses and heminodes, respectively.

To send information about sound to the brain, cochlear inner hair cells (IHCs) excite the auditory nerve by exocytosis of glutamate onto AMPA-type receptors at afferent ribbon synapses (Ruel et al., 1999; Glowatzki and Fuchs, 2002; Figure 1A). Each auditory nerve fiber (ANF, or spiral ganglion neuron) is driven by a single, large afferent synapse where the post-synaptic density (PSD) measured with electron microscopy is ~ 850 nm in length, on average, and the two-dimensional surface area ranges 0.1–1.5 μm^2^ [cat: Liberman, 1980; Merchan-Perez and Liberman, 1996; mouse: Meyer et al., 2009; see also Payne et al. (2021) in this special issue]. Glutamatergic synapses in the brain are considerably smaller, where PSDs are ~ 300 nm in length, on average, and most have surface area <0.1 μm^2^ (Aoki et al., 2001; Qu et al., 2009; Santuy et al., 2018). Assuming a packing density of 900 AMPA receptors per μm^2^ (Momiyama et al., 2003), these cochlear synapses would contain up to ~1,350 receptors per afferent terminal, whereas a typical central PSD of 0.1 μm^2^ would have only ~ 90 receptors. The PSD of the ANF supports excitatory postsynaptic currents (EPSCs) hundreds of picoamps in amplitude in response to each presynaptic release event. Spikes are evoked at the nearby heminode, one EPSC at a time, at sustained rates of hundreds of spikes per second (Sachs and Abbas, 1974; Siegel, 1992; Rutherford et al., 2012). The spike-generating heminode of each ANF is separated from its afferent synapse by only ~20 μm of dendrite with a diameter <1 μm (Hossain et al., 2005; Kim and Rutherford, 2016; Figure 1B, see yellow and magenta asterisks positioned at the ribbon synapses and heminodes, respectively). Such tight spatial coupling between synapse and spike generator may be essential for precise encoding of acoustic information represented in the temporal pattern of synaptic transmission (Rutherford and Moser, 2016). At the same time, high activity rates such as those that occur during acoustic overexposure may render this unmyelinated small-volume segment of the ANF within the organ of Corti vulnerable to ionic imbalance (Figure 1C). Immediately after a 90-min exposure to 4–8 kHz octave band noise at 109 dB SPL, we observed some dendritic fragmentation between the ribbon synapses in the inner spiral plexus (ISP) and the heminodes at the habenula perforata (HP, vertical arrowheads), consistent with synapse loss and osmotic damage seen after synaptopathic noise exposure in confocal and electron microscopy that can be blocked with the glutamate receptor antagonist kynurenic acid (Robertson, 1983; Puel et al., 1998; Furman et al., 2013; Hickman et al., 2020).

Severe acoustic trauma can kill hair cells and permanently elevate hearing thresholds. Moderate exposures that only temporarily elevate hearing thresholds, with no immediate loss of hair cells, may nonetheless result in immediate damage to or loss of afferent synapses (Kujawa and Liberman, 2009; Lin et al., 2011). Noise-induced cochlear synaptopathy is defined as synapse loss or dysfunction as a result of acoustic trauma (Moser and Starr, 2016; Liberman, 2017), and is associated with reduction in the amplitude of wave-I of the auditory brainstem response (ABR) while otoacoustic emissions and auditory thresholds are unchanged. The auditory nerve compound action potential (CAP) is an analog of ABR wave-I, a metric of cochlear output in animals including human (Relkin et al., 1995; Murnane et al., 1998). Noise-induced cochlear synaptopathy triggers or accelerates primary neuronal degeneration that precedes hair cell loss and, in mice, is associated with an increased rate of subsequent age-related permanent threshold shift (Kujawa and Liberman, 2006; Stamataki et al., 2006). The pattern of ANF degeneration seen in the organ of Corti and osseous spiral lamina of animal models following synaptopathic noise exposure, and cell death in the spiral ganglion with hair cells intact, is seen also in human postmortem cochleae (Viana et al., 2015; Wu et al., 2019). This noise-induced cochlear synaptopathy is a form of excitotoxicity that appears to depend on synaptic release of glutamate from inner hair cells (IHCs), as demonstrated in the vesicular glutamate transporter type-3 (Vglut3) knockout mouse (Kim et al., 2019). In the guinea pig, synaptopathic noise exposure resulted in synaptic reorganization, impaired temporal processing, and selective reduction in the proportion of ANFs with the low-spontaneous rate/high-threshold phenotype thought to be important for the encoding of suprathreshold sounds in noisy environments (Furman et al., 2013; Shi et al., 2013; Hickman et al., 2020). Higher doses of noise can result in cochlear synaptopathy accompanied by immediate loss of outer hair cells and permanent threshold shifts (Fernandez et al., 2020). A better understanding of ANF excitability could enable development of therapies for synaptopathy by targeting excitotoxicity; however, the molecular mechanisms of glutamate excitotoxicity are unclear.

AMPARs are tetramers comprised of combinations of subunits GluA1−4 in the brain. Curiously, the mature cochlea appears to express only GluA2−4 (Niedzielski and Wenthold, 1995; Matsubara et al., 1996; Parks, 2000; Shrestha et al., 2018). In the adult brain, the overwhelming majority of AMPARs contain an RNA-edited form of the GluA2 subunit rendering the channel relatively impermeable to Ca^2+^, resulting in Ca^2+^-impermeable AMPARs (CI-AMPARs; Sommer et al., 1991; Higuchi et al., 1993). AMPARs lacking edited GluA2 are called Ca^2+^-permeable AMPARs (CP-AMPARs) because they have greater permeability to Ca^2+^ and larger overall ionic conductance, carried mainly by Na^+^ (Hollmann et al., 1991; Geiger et al., 1995). The expression of GluA2-lacking CP-AMPARs is downregulated in the developing brain (Pickard et al., 2000; Kumar et al., 2002; Henley and Wilkinson, 2016). However, they persist in some neurons of the auditory brainstem where they may be important for fast transmission of acoustic signals (Raman et al., 1994; Trussell, 1997; Wang et al., 1998; Gardner et al., 1999, 2001; Lawrence and Trussell, 2000; Sugden et al., 2002; Wang and Manis, 2005; Youssoufian et al., 2005; Lujan et al., 2019). Some studies have implicated CP-AMPARs in mechanisms of long-term potentiation, long-term depression, and homeostatic scaling; however, this remains controversial as several recent studies failed to find specific involvement of CP-AMPARs in these processes (for review, see Henley and Wilkinson, 2016). In any case, aberrant regulation of CP-AMPARs has been implicated in many disorders of the central and peripheral nervous system in which excessive activity results in oxidative stress and neurodegeneration (Cull-Candy et al., 2006; Kwak and Weiss, 2006; Liu and Zukin, 2007; Weiss, 2011).

The extent to which CP-AMPARs exist in the ear is not entirely clear. They may be downregulated over development in the rat cochlea (Eybalin et al., 2004), as in the brain. In rodents, cochlear afferent synapses are typically quantified with confocal microscopy by counting the juxtaposition of immunohistofluorescence of the presynaptic ribbon paired with postsynaptic GluA2 (Khimich et al., 2005; Meyer et al., 2009; Liberman et al., 2011; Figure 1). All cochlear afferent synapses appear to express GluA2, leading to the supposition that CP-AMPARs are not present in the cochlea, if cochlear GluA2 RNA is edited as in the brain. Although GluA2 may be present at every synapse, each cochlear afferent synapse is thought to contain hundreds to thousands of AMPARs. We hypothesize that a subset of the AMPARs at each synapse lack GluA2 in the tetramer. High-resolution confocal microscopy of cochlear afferent synapses has suggested GluA2-lacking nanodomains within the large PSDs on ANFs (Sebe et al., 2017; Hu et al., 2020). Moreover, chronic local delivery of an antagonist (IEM-1460), selective for CP-AMPARs at low μM concentrations, prevented noise-induced glutamate excitotoxicity in the mouse cochlea (Hu et al., 2020). This study suggested GluA2-lacking CP-AMPARs could be therapeutic targets at mature cochlear afferent synapses; however, the concentration of IEM-1460 in perilymph was not measured, thus the selectivity of the blockade was uncertain.

IEM-1460 and IEM-1925 are dicationic compounds that are non-competitive open-channel blockers selective for the pore of GluA2-lacking CP-AMPARs (Magazanik et al., 1997; Tikhonov et al., 2000). A non-competitive blocking mechanism should not interfere with glutamate binding to the ligand-binding domain of AMPA, NMDA, kainate, or metabotropic glutamate receptors, thus reducing the potential for off-target effects at other glutamate receptors. Open-channel blockers are use-dependent, requiring channel activation for access to the pore region of the transmembrane domain, thus the blocking effect develops exclusively in the presence of an agonist (Tikhonova et al., 2008; Twomey et al., 2018). Here, in guinea pig, with intracochlear perfusion of defined concentrations of the compound in artificial perilymph, we measured the immediate concentration-dependent effects of IEM-1460 on ANF activity. Further, with systemic dosing *in vivo* and with cell-based assays we assessed pharmacokinetics, metabolic stability, and toxicity of IEM-1460 and IEM-1925, both of which are known to be selective CP-AMPAR antagonists.

## Materials and Methods

### Animals

This study utilized 21 NIH-strain, pigmented guinea pigs weighing from 400 to 650 g, in accordance with policies and protocols approved by the Institutional Animal Care and Use Committee at Washington University in St. Louis (20180133, 20190035). Animals were anesthetized with intraperitoneal (IP) injection of 100 mg/kg sodium thiobutabarbital (Inactin, Sigma, St. Louis, MO). Once anesthetized, the fur from the head and neck was shaved and a tracheal cannula was placed. The animal was maintained on 0.8–1.2% isoflurane in oxygen using a mechanical ventilator. The ventilator's tidal volume was adjusted to maintain a 5% end-tidal CO_2_ which was monitored with a CapnoTrue AMP (Bluepoint Medical, Zevenaar, The Netherlands). Body temperature was maintained at 38 degrees C with a DC-powered heating blanket-rectal thermometer (Model 50-7079; Harvard Apparatus). Heart rate and oxygen saturation were monitored with a pulse oximeter (Surgivet, Waukesha, WI). The auditory bulla was exposed ventrally for placement of recording electrodes. The jugular vein was cannulated to maintain hydration with 0.5 mL per hour of Lactated Ringer's solution. To prevent middle ear muscle contractions, vecuronium bromide (0.2 mg/kg) was administered immediately prior to data collection and every 1.5 h thereafter.

### Immunostaining of Cochlear Synapses

Following cardiac perfusion of 4% PFA in 0.1 M phosphate buffer and cervical dislocation, the temporal bone was immediately harvested, and the cochlea was isolated. Each cochlea was post-fixed for 20 min at 4°C in a dish with 4% PFA, where a small fenestration was made in the round window, oval window, and the apex of each cochlea to gently flush with 1 mL of fixative. Each cochlea was then decalcified in 10% EDTA at room temperature for 6 h, exchanging EDTA every 2 h. The decalcified outer bone was removed to reveal the underlying cochlear spiral. Small pieces of cochlear turns were placed in buffer overnight at 4°C in Triton X-100 with donkey serum. The following primary antibodies were used: anti-C-terminal binding protein-2 to label presynaptic ribbons (CtBP2 mouse IgG1, 1:300, BD Biosciences #612044, RRID AB_399431); anti-AMPA receptor subunit-3 to label post-synaptic densities (GluA3 goat, 1:200, SCBT, RRID AB_2113895); anti-myosin VII-A to label inner hair cell cytoplasm (Myo7a rabbit, 1:400, Fisher Scientific #NC0165634, RRID AB_2553946); anti-vesicular glutamate transporter-3 (Vglut3 goat, 1:400, SCBT RRID AB_2187701); anti-adenosine triphosphate-dependent sodium/potassium exchanger-α3 to label neuronal membranes (NaKATPase mouse IgG1, Life Tech MA3-915, RRID AB_2274447); anti-pan voltage-gated sodium channel-α1 to label heminodes and nodes (NaV1 mouse IgG2a, AB Inc. 75-405, RRID AB_2314861). The primary antibodies were labeled with species-appropriate secondary antibodies conjugated to Alexa Fluor 488, 555, or 647 (1:200, Life Technologies Corp.). Digital images were obtained using a Zeiss LSM700 confocal microscope with 63× oil immersion objective, 1.4 N.A., with pixel size of 50–100 nm in X and Y, and Z step size of 0.3–0.5 μm.

### Stimulus Generation and Recording for Experiments With Intracochlear Drug Delivery

The head was stabilized in a head-holder with a solid ear bar on the left side and hollow ear bar on the right side, a bite bar, and a snout clamp. Sound calibration, stimulus generation, and recording methods have been detailed previously (Lichtenhan et al., 2016a, 2017). Briefly, cochlear function measurements were made using MATLAB software (The MathWorks, Natick, MA) and the software utility Playrec. The stimulus was first generated in MATLAB, digitized at 96 kHz, presented to a 24-bit sound card (RME: Fireface802; Sweetwater, Fort Wayne, IN), and then routed to an acoustic probe system (ER-10X; Etymotic Research, Fort Wayne, IN). The ER-10X microphone was calibrated using a reference microphone (1/8” GRAS type 40P) and a custom-made coupling system was used for setting stimulus levels. Sound was presented through the hollow ear bar on the right side via the ER-10X microphone probe.

Cochlear output was recorded as a field potential between a Ag/AgCl ball electrode in the cochlear round window niche and a platinum needle electrode at the vertex of the cranium. Responses to 20 tone bursts (10 with reversed polarity) were averaged for each CAP measurement at a given frequency and level. The CAP audiogram was measured (1–22 kHz, 1/4 octave steps) at the beginning and end of the experiment to determine CAP threshold as a function of tone-burst frequency. Prior to and immediately after IEM-1460 perfusions via the apical cochlear fenestration (see below), CAPs were evoked by tone bursts at selected frequencies and levels. Measurements were repeated at 1- to 2-min intervals to monitor cochlear output and to ensure response stationarity for at least 25 min prior to drug delivery. Data with baseline changes >20% were excluded. The round window niche was monitored for accumulation of fluid.

### Stimulus Generation and Recording for Experiments With Systemic Drug Delivery

As described for local delivery, cochlear output was measured similarly for systemic delivery of IEM-1460 and IEM-1925. Complete details have been previously published (Salt et al., 2013). Briefly, acoustic tone bursts were applied in a closed sound system. The plastic extension tube of the acoustic assembly (Etymotic Research, ER-10 C) was coupled to the end of the hollow ear bar with the tube tip positioned ~1.5 mm from the tympanic membrane. Stimulus generation and data collection were performed with Tucker Davis System 3 hardware controlled by a custom written program (Microsoft Visual Basic). Stimulus waveforms were routed through a programmable Tucker Davis PA5 attenuator and HB7 headphone amplifier. Stimuli were calibrated from 0.125 to 26 kHz in 1/4 octave steps. A CAP audiogram was obtained at the beginning and end of the experiment. Up to 60 min of baseline audiometric data were obtained prior to drug delivery. Data with baseline changes >20% were excluded. A 13-level tone probe series, at selected frequencies, was repeated at 2-min intervals. Distortion product otoacoustic emissions (DPOAEs) were measured using an ER10C probe system. The two primary tone frequencies were set at 6 kHz (f1) and 7.4 kHz (f2), for a f2/f1-ratio of 1.23, with levels of 65 dB SPL (L1) and 55 dB SPL (L2).

### Electrophysiological Data Analysis and Statistics

At each probe frequency, CAP peak-to-peak amplitudes were measured from the first trough (N1) to the first peak (P1). Latencies were measured to the trough of N1. Cochlear output was compared before and after intraperitoneal drug delivery. The data were tested for normality using a Kolmogorov-Smirnov test. For CAP amplitude and latency, significance was assessed with the Mann-Whitney *U*-test. The Wilcoxon signed rank test was used to assess CAP threshold. Friedman test was used to compare concentrations of drug between perilymph and CSF. A *p* < 0.05 was considered statistically significant. A Bonferroni correction was used when performing multiple comparison testing. All statistical tests were performed with IBM SPSS Statistics Version 27 (SPSS Inc., Chicago, IL, United States).

### Intracochlear Drug Delivery

The method of apical intracochlear drug delivery has previously been well-detailed (Lichtenhan et al., 2016a). IEM-1460 or kainic acid (KA) in artificial perilymph (in mM: 127.5 NaCl, 3.5 KCl, 25 NaHCO_3_, 1.3 CaCl_2_, 1.2 MgCl_2_, 0.75 NaH_2_PO_4_, and 11 glucose) was injected into scala tympani from a glass pipette that was sealed into the cochlear apex. We refer to this method, below, as the apical perfusion technique. To insert the injection pipette, the mucosa on the bone of the fourth cochlear turn was removed with a moist, cotton wrapped applicator. A thin layer of cyanoacrylate glue was then applied to the dried area (no. 101; Permabond) followed by a layer two-part silicone elastomer to make a hydrophobic cup-shaped dike (Kwik-Cast; World Precision Instruments). A fenestration was then made through the cup-shaped elastomer (~100–150 μm) with a 30° pick (Storz N170580, 1/3 mm 30° oval window pick, Bausch & Lomb Surgical, St. Louis, MO, USA). The size of the fenestration was just large enough to insert a glass pipette (~50 μm diameter tip) which was then sealed into the fenestration with the application of additional cyanoacrylate glue to form a seal and prevent the possible leakage of perilymph. Solutions in the injection pipette were programmatically driven by a 50 or 100 μL gastight syringe (1710TLL; Hamilton Syringe) mounted on a computer-controlled ultrapump (UMP3, World Precision Instruments, Sarasota, FL). Injection of drug solution at a rate of 500 nL/min for 15 min drives drug solution toward the cochlear aqueduct, filling scala tympani from the apex to the base, controlling drug concentration by minimizing physiological dilution. Here, the apical perfusion technique was applied for 15 min, with the scala tympani being fully loaded within 12 min. Response waveforms were recorded immediately prior to apical drug perfusion to establish a baseline, throughout perfusion which took 6 min, and then immediately after perfusion, from a round window electrode. Cochleae were prepared for fixation immediately following perfusion.

### Intraperitoneal Drug Delivery

After at least 30 min of baseline data, 1 mL of Lactated Ringer's was injected into the peritoneal space of the guinea pig as a vehicle control. Then, 13.5 mg/kg IEM-1460 or 8 mg/kg IEM-1925 in Lactated Ringer's was injected into the peritoneal space of the guinea pig. The entire experiment took ~3 h to complete: 1 h for anesthetic induction and surgery, 30 min for sound calibration and CAP threshold measurements, and 2 h for drug delivery, DPOAE and CAP measurements. After collection of perilymph and CSF, the animal was euthanized via saturated KCl delivered directly into the jugular vein.

### Collection of Blood, Perilymph, and CSF After IP Drug Injection

General surgical scissors were used to clip the toenail at a 45° angle to collect blood with a premarked glass capillary tube during the baseline period and after drug injection. The exact volume was determined by measurement of the fluid meniscus in a calibrated capillary tube. Sampling of perilymph from the cochlear apex was performed to measure drug concentrations once the CAP amplitude had decreased, suggesting that there had been drug penetration within the cochlea. Complete details used to perform apical fluid sampling are presented elsewhere and are only briefly provided here (Mynatt et al., 2006). Similar to the apical cochlear fenestration used to deliver drugs (described above), the mucosa on the cochlea was cleared from two apical turns using a small cotton swab, and the bone at the apex was allowed to dry. A droplet of thin cyanoacrylate adhesive was applied and cup formed over the apex with silicone adhesive. A small perforation (50–100 μm diameter) was then made in the bone over scala vestibuli at the apex using a 30° pick. Upon perforation, fluid immediately started emerging, driven by intralabyrinthine pressure from CSF, and formed a bead within the hydrophobic silicone cup. A calibrated 5 μL capillary micropipette (VWR #5342-706, S. Plainfield, NJ) was held by hand in contact with the fluid at the apex, so that fluid was drawn into the capillary in ~30 s. The exact volume was determined by measurement of the fluid meniscus. Perilymph from the opposite ear was sampled prior to euthanizing the animal, then ~2 μL of CSF was aspirated by inserting a micropipette through the dura mater into the cisterna magna. After the fluid meniscus from the capillary tube was measured, the capillary tube was then flushed with 25.0 μL 50:50 methanol-water solvent to ensure that all the sample was collected.

### Liquid Chromatography and Mass Spectroscopy (LC/MS)

**Figure 2:**
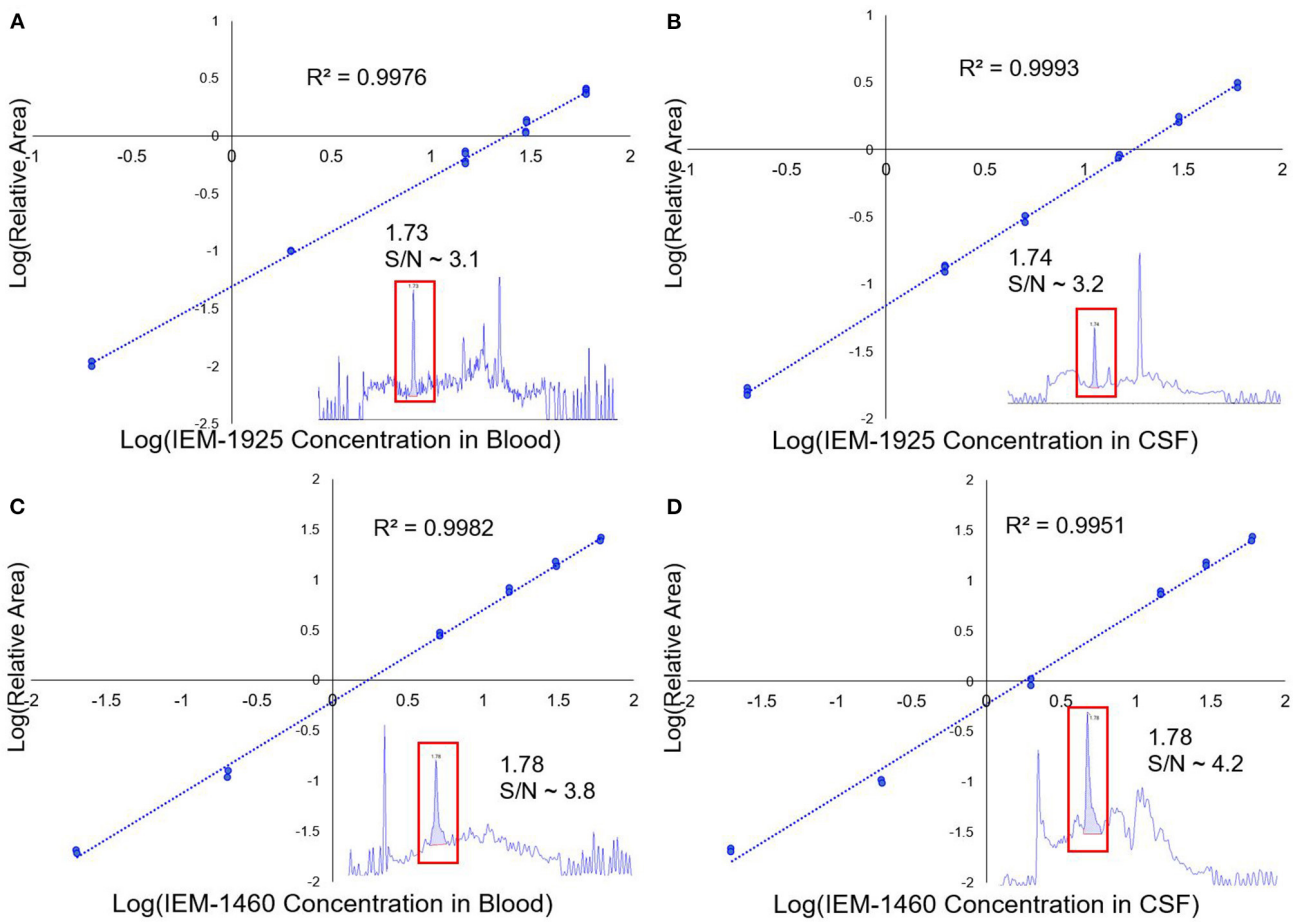
LC/MS calibration curves for IEM-1925 and IEM-1460. **(A)** The LC/MS calibration curve showing the relative area of IEM-1925 to internal standard (verapamil) in blood is plotted against IEM-1925 concentration. Replicates for each concentration were obtained with duplicate injections. The 1 ng/mL standard in blood chromatogram with S/N ~ 3.1 is shown in the inset that eluted at 1.73 min. **(B)** Similarly, LC/MS calibration curve showing IEM-1925 in CSF matrix is plotted against the internal standard. The 1 ng/mL standard in CSF with S/N ~3.2 is shown in the inset eluting at 1.74 min. **(C)** IEM-1460 calibration curve in blood with inset showing 1 ng/mL IEM-1460 in blood with S/N ~3.8 eluting at 1.78 min. **(D)** IEM-1460 calibration curve in CSF with inset showing 1 ng/mL IEM-1460 in CSF with S/N ~ 4.2 eluting at 1.78 min.

The concentration of IEM-1460 in perilymph, blood, and CSF was analyzed using LC-MS (Confluence Life Sciences, St. Louis, MO). LC-MS-grade acetonitrile, LC-grade methanol, formic acid, sodium hydroxide 0.1 M, perfluoroheptanoic acid, formic acid, and hydrochloric acid were purchased from Sigma-Aldrich (USA). Deionized water (R > 18 MΩ/cm, TOC <10 ppb) was used throughout all these experiments. IEM-1460 and IEM-1925 were purchased from Tocris Bioscience (Bristol, UK). A 10 mM stock solution was made for both IEM-1460 and IEM-1925 in 50% aqueous methanol. For calibration of the specific compound concentration in the tested matrix (i.e., plasma or CSF/perilymph), a set of standard solutions were prepared by diluting concentrated stock solutions with 50% aqueous methanol into guinea pig plasma matrix or artificial perilymph (in mM: 127.5 NaCl, 3.5 KCl, 25 NaHCO_3_, 1.3 CaCl_2_, 1.2 MgCl_2_, 0.75 NaH_2_PO_4_, and 11 glucose) to have a set of standards from 1 to 300 ng/mL. Figure 2 shows the calibration curves and chromatograms in both blood and CSF for compounds IEM-1460 and IEM-1925. Calibration standard samples using verapamil were prepared for each batch of samples ranging from 1 to 300 ng/mL and injected at both the beginning and end of the sample sequence. The methodology for LC-MS for blood has previously been described (Da Silva et al., 2016; Guiraud et al., 2017). Separation and analysis were performed on a LC instrument (Exion LC AD, AB SCIEX, USA) coupled to a Sciex QTRAP5500 with TurboV Ion Source (SCIEX, Concord, ON, Canada) equipped with a heated electrospray ionization (H-ESI) source. Chromatographic separation was obtained using a gradient elution on a reversed-phase Kinetex F5 (Phenomenex, Aschaffenburg, Germany) column (2.1 × 50 mm), packed with 1.7 μm diameter particles. The injection volume was 5 μL, and the total run time for this analysis was 6 min.

Immediately after collection of the guinea pig samples of blood, CSF, and perilymph obtained following IP injection, the samples were diluted in 25 μL of 50:50 methanol-water solvent and were subsequently stored at −80°C. Prior to LC/MS analysis, perilymph, blood, and CSF samples were thawed and vortexed for 2 min. Approximately 25 μL of the diluted sample was transferred into a 0.5 mL microcentrifuge tube. The tube was then placed into a multitube vortexer for 15 min at 1,350 rpm at 4°C and then centrifuged at 14,800 rpm for 10 min. The supernatants were then pipetted and filtered through a 0.22 μm filter and placed into a 96-well plate for LC-MS analysis. To assess the matrix effects of the column, a sample containing the internal standard analyte added to the extracted matrix and another sample containing the internal standard analyte in the mobile phase solvent were used. The same concentration of the internal standard analyte was used in both samples. Matrix effect values were calculated by (%) = B/A ^*^ 100 (A = mean of external solution peak area, B = post-extraction sample peak area). The LC/MS procedure was validated using specificity and selectivity, linearity, trueness, precision, recovery, limit of detection (LOD), and limit of quantification (LOQ). The mass spectrum profile and retention time of pure standards were used to assess specificity and selectivity by comparing this value to unspiked CSF and plasma samples. LOD was assessed by ensuring that the samples were at least three times higher than the signal-to-noise ratio (SNR) and LOQ was assessed by ensuring that analytes were 10 times higher than the SNR. Calibration curves were constructed for each concentration level by calculating the chromatographic peak area ratio of the analyte in comparison to the internal standard area. Repeatability was assessed by spiking the plasma and perilymph standard solutions with low, medium, and high levels of IEM. Six replicates were performed for each standard solution by the same operator on three separate occasions.

### *In vitro* Microsomal Stability Assay

The stability of IEM-1460 in the presence of guinea pig liver microsomes (BioIVT, PN: X008070) or IEM-1925 in the presence of human (BioIVT, PN: M00101), mouse, or guinea pig liver microsomes was evaluated (Paraza Pharma, Inc., Montreal, Quebec, Canada). Both IEM-1925 and IEM-1460 were incubated with microsomes at 37°C for a total of 45 min. Working solutions were prepared in 100 mM potassium phosphate buffer and the final reaction was performed at pH 7.4 in 100 mM potassium phosphate buffer containing 0.5 mg/mL liver microsomal protein. Phase I metabolism was assessed by adding NADPH to a final concentration of 1 mM and collecting samples at time points 0, 5, 15, 30, and 45 min. All collected samples were quenched 1:1 with ice-cold stop solution (1 μM labetalol and 1 μM glyburide in acetonitrile) and centrifuged to remove precipitated protein. Resulting supernatants were further diluted 5-fold with water. Samples were analyzed by LC-MS/MS using a Thermo Vanquish UPLC and a Thermo Quantis triple quadrupole mass spectrometer. Analytes were injected into a Phenyl column and chromatographed using a reverse phase gradient with 0.1% formic acid in water and 0.1% formic acid in 20/80 Isopropanol/Acetonitrile mobile phases. Reference compounds were also assessed to ensure accuracy and precision of experimental protocol: imipramine and verapamil for human liver microsomes, diphenhydramine and verapamil for mouse liver microsomes, and warfarin and verapamil for guinea pig liver microsomes. Calculations for half-life, *in-vitro* clearance, and percentage of hepatic blood flow were performed using Microsoft Excel. Half-life was determined from a plot of the natural logarithm of the peak area ratio (remaining compound peak area/internal standard peak area) against time.

### Radioligand Binding Assays

Binding assays were conducted to evaluate the affinity of test compounds IEM-1460 and IEM-1925 (Eurofins Scientific, Inc.) for 15 targets: the human alpha-1 adrenergic (A1) receptor, histaminergic (H)-2 and−3 receptors, and muscarinic acetylcholine receptors-1 to−5 in transfected CHO cells by radioligand binding. The neuronal α4β2 and neuronal α7 cholinergic acetylcholine receptors were tested in transfected SH-SY5Y cells. The histaminergic-1 and−4 receptors were tested in transfected HEK-293 cells. Ion channel testing for AMPA and N-methyl-D-aspartate (NMDA) was performed in cells from the rat cerebral cortex. For alpha-1 adrenergic receptor (Townsend-Nicholson and Schofield, 1994), cell membrane homogenates (~23 μg protein) were incubated for 1 h at 37°C with 1 nM of [3H]DPCPX in the absence or presence of IEM-1460 or IEM-1925 in a buffer containing the following in mM: 25 HEPES/KOH (pH 7.0), 100 NaCl, 1.5 CaCl_2_, 1 MgSO_4_, and 0.2 g/L 1.10 phenanthroline and 0.1% BSA. Non-specific binding was determined in the presence of 1 μM of DCPX. Following incubation, the samples were filtered rapidly under vacuum through glass fiber filters (GF/B, Packard), presoaked with 0.3% PEI and rinsed several times with ice-cold 50 mM Tris-HCl using a 96-sample cell harvester (Unifilter, Packard). The filters were dried and then counted for radioactivity in a scintillation counter (Topcount, Packard) using a scintillation cocktail (Microscint 0, Packard). The results are expressed as a percent inhibition of the control radioligand specific binding: 100-[(measured specific binding)/(control specific binding) × 100]. IEM-1460 or IEM-1925 was tested at 1 μM to determine the percent inhibition of binding when compared to the reference compound (a known agonist) for each specific receptor. These steps were repeated for H1 (Smit et al., 1996), H2 (Leurs et al., 1994), H3 (Lovenberg et al., 1999), H4 (Liu et al., 2001), M1-2 (Dörje et al., 1991), M3 (Peralta et al., 1987), M4-5 (Dörje et al., 1991), N-α4β2 (Gopalakrishnan et al., 1996), N-α7 (Sharples et al., 2000), AMPA (Murphy et al., 1987), and NMDA (Sills et al., 1991) receptors.

## Results

### Destruction of Ribbon Synapses and Afferent Terminals With Intracochlear Perfusion of Glutamate Receptor Antagonist

**Figure 3:**
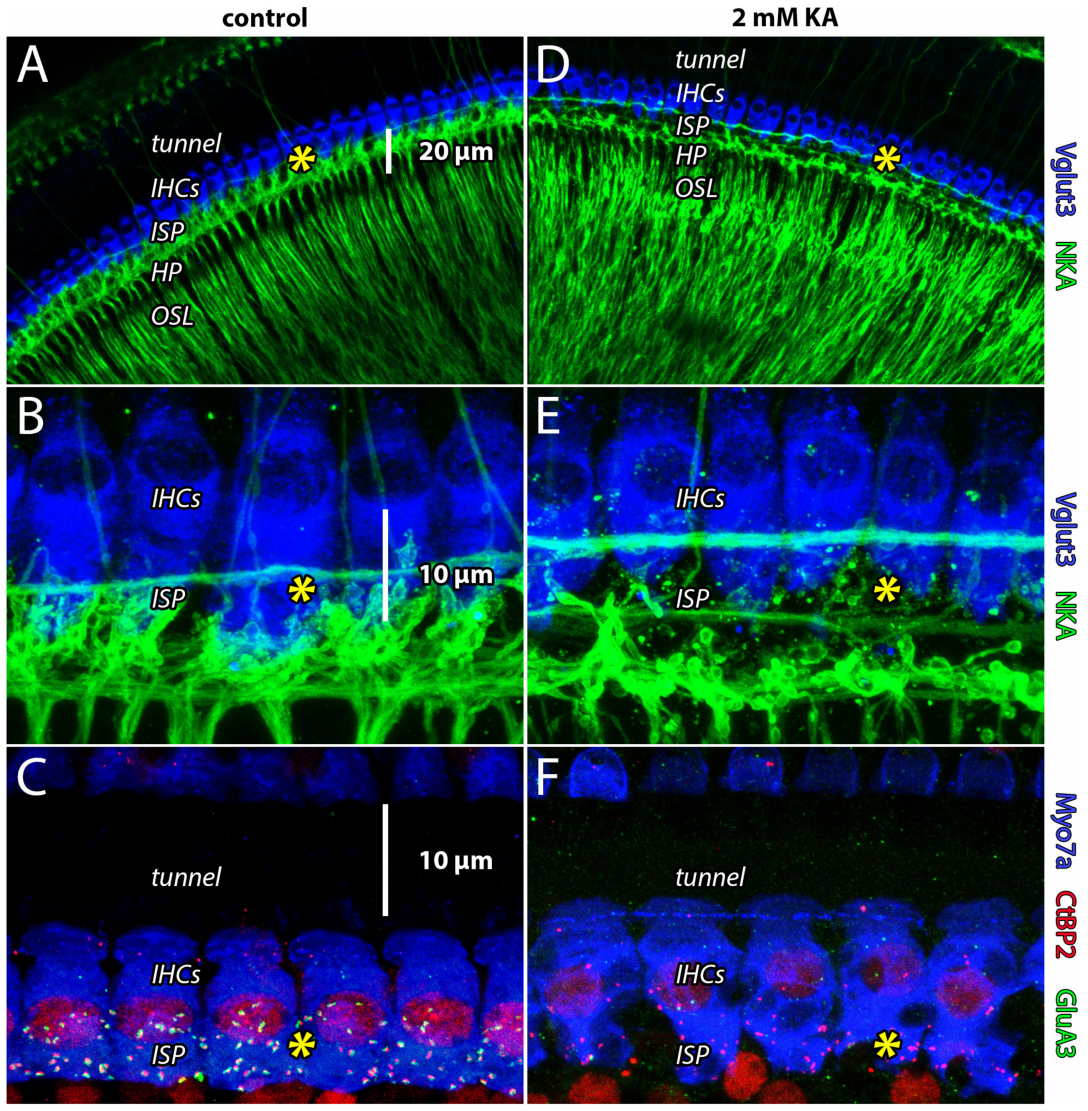
Apical cochlear perfusion of kainic acid lesions ANF dendrites throughout the cochlear spiral. **(A,B)** Whole-mount preparation from the apical turn (turn four) of guinea pig cochlea. Inner hair cells (IHCs) are labeled with anti-Vglut3 (blue); Type-I auditory nerve fibers innervating IHCs in the inner spiral plexus (ISP) and tunnel-crossing medial olivocochlear efferent neurons are labeled with anti-Na^+/^K^+^-ATPase (green). In all panels, yellow asterisk indicates location of ribbon synapses in the ISP. **(C)** Whole-mount preparation from the basal turn (turn one). In control samples, presynaptic ribbons (anti-CtBP2, red) of IHCs (anti-Myosin7a, blue) are paired with postsynaptic AMPA receptors (anti-GluA3, green) in the ISP. Anti-CtBP2 also labels cell nuclei. Perfusion of artificial perilymph into scala tympani from the cochlear apex resulted in images like those taken from naïve controls, showing dense innervation in the neuropil of the organ of Corti under IHCs (in the inner spiral plexus, ISP). **(D–F)** Disintegration of synapses from the cochlear apex (turn four) to the base (turn one) confirms the apical perfusion solution accessed the scala tympani all the way down the length of the cochlea.

Previous studies perfused the cochlea with glutamate receptor agonists and antagonists through cochleostomy in the basal turn of the cochlea (Puel et al., 1994). To demonstrate the effectiveness of our apical injection technique to target the entire cochlea from apex to base, we perfused the guinea pig cochlea with artificial perilymph with or without the AMPA/kainate receptor agonist kainic acid (KA), followed by immunohistofluorescence and confocal microscopy (Figure 3). Perfusion of artificial perilymph into scala tympani from the cochlear apex resulted in images like those taken from naïve controls, showing dense innervation in the neuropil of the organ of Corti under IHCs in the ISP (Figures 3A–C, see yellow asterisks). Perfusion of artificial perilymph containing 2 mM KA resulted in a void of innervation in the ISP and ribbon synapse disintegration (Figures 3D–F, see yellow asterisks), consistent with terminal swelling and dendrite retraction seen with chemical lesion in electron microscopy (Puel et al., 1994). Disintegration of synapses in turn one (Figure 3F) confirms that the apical injection method perfuses the scala tympani all the way to the base of the cochlea.

### Dose-Dependent Reduction of Auditory Nerve Activity With Intracochlear Perfusion of Selective CP-AMPAR Antagonist IEM-1460

Previously, we blocked noise-induced synapse loss and concomitant reduction of ABR wave-I with chronic intracochlear delivery of IEM-1460 via a cannula inserted through the round window, connected to an osmotic minipump implanted in the middle ear of mice (Hu et al., 2020). The concentration of IEM-1460 eluted from the minipump was 500 μM; however, the intracochlear concentration of the diluted compound was not known. At high concentration, IEM-1460 will block GluA2-lacking and GluA2-containing AMPARs (IC_50_ = ~500–1,100 μM on GluA2-containing AMPARs) (Magazanik et al., 1997; Buldakova et al., 1999). At low concentration, it is selective for GluA2-lacking receptors. Cells expressing GluA2-lacking AMPARs exhibit high sensitivity to IEM-1460 (IC_50_ = ~1–3 μM). Cells expressing a combination of CP-AMPARs and Ca^2+^-impermeable receptors (CI-AMPARs) have intermediate sensitivity.

Auditory nerve activity is triggered by the activation of AMPARs by glutamate. Thus, to interrogate AMPAR subunit composition in the guinea pig, we asked if IEM-1460 could reduce sound-evoked auditory nerve activity at concentrations consistent with antagonism of GluA2-lacking CP-AMPARs. First, we used the apical perfusion technique to test the effect of different concentrations of IEM-1460 on cochlear responses to sound. When a tone burst is applied to the ear, synchronous exocytosis of glutamate from IHC ribbon synapses triggers synchronous depolarization of ANFs through activation of AMPARs, triggering action potentials. The amplitude of the auditory nerve field potential, the CAP, reflects the number of contributing ANFs (Earl and Chertoff, 2010).

**Figure 4:**
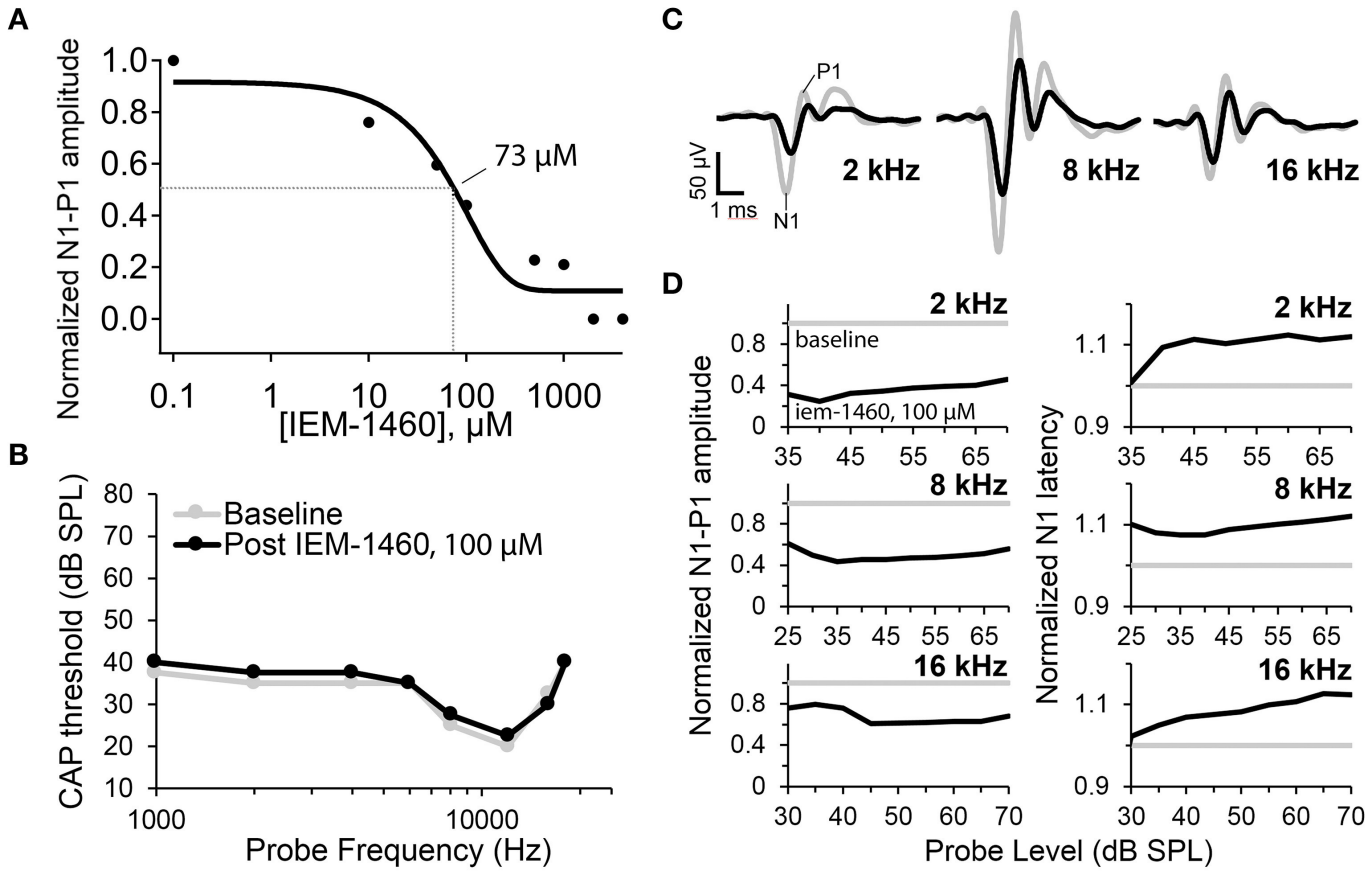
Auditory nerve compound action potential (CAP) measurements before and after cochlear perfusion with IEM-1460. **(A)** IEM-1460 dose-response inhibition curve shows relative reduction in CAP amplitude with increasing concentration of IEM-1460 varying from 0.1 to 4,000 μM (*N* = 6 female guinea pigs, 8–9-weeks-old). Probe level was 80 dB SPL. Fractional reduction from baseline was averaged for responses to 2 and 16 kHz tones. IC_50_ = ~ 73 μM. **(B)** CAP thresholds across frequencies before and after perfusion of 100 μM IEM-1460 in artificial perilymph into scala tympani from an injection pipette sealed to the cochlear apex. **C)** CAP waveforms in response to 70 dB SPL tone bursts at 2, 8, and 16 kHz. Gray traces were recorded before perfusion, and black traces were recorded after perfusion of 100 μM IEM-1460 in artificial perilymph. **(D)** Baseline normalized CAP amplitudes (N1-P1, left) and N1 latencies (right) as a function of probe level for the three probe frequencies. Perfusion with 100 μM IEM-1460 reduced CAP amplitude and increased CAP latency.

At 10 μM IEM-1460, the amplitude of the tone-evoked CAP was reduced by ~25%. With increasing concentration, the CAP progressively decreased. For 80 dB SPL tones at 2 and 16 kHz, on average, the CAP amplitude was reduced by ~55% with 100 μM IEM-1460 in the perfusion pipette (Figure 4A). Relative to the pre-drug baseline recording, the reduction in CAP amplitude with 100 μM IEM-1460 was greater for lower-frequency tones (~ 60% at 2 kHz) than for higher-frequency tones (~30% at 16 kHz) (Figures 4C,D, left). Although we don't know the reason for this difference, it would be expected if either the mechanical disturbance of the injection was greater or if the IEM-1460 concentration was greater near the perfusion pipette in the apex, and/or if AMPARs of apical ANFs had less GluA2 and thus greater Ca^2+^-permeability. Relative to pre-drug measures, CAP latencies increased by ~10% (additional delay of ~0.3 ms) across tone levels and frequencies in the presence of 100 μM IEM-1460 (Figure 4D, right). There was a trend toward longer relative latency shifts in response to higher-level tones than lower-level tones.

The dose-response inhibition plot for relative CAP reduction with increasing concentration of IEM-1460 was fit with a Boltzmann sigmoidal equation to obtain the concentration for a 50% CAP reduction (IC_50_) of 73 μM. Thus, at ~73 μM IEM-1460, the AMPAR-mediated excitatory postsynaptic current (EPSC) of the guinea pig cochlea was antagonized enough to reduce the amplitude of the auditory nerve field potential by 50%. Assuming linear summation, this suggests that ~ 50% of ANFs failed to spike at sound onset under this condition. We note this 50% reduction in the CAP does not imply a 50% reduction of the EPSC (see Discussion).

For a given tone frequency, the sound level needed to evoke a 10 μV CAP waveform amplitude is considered the “threshold level” (dB SPL). CAP thresholds correlate very closely with behavioral thresholds for auditory perception in rodents (Dallos et al., 1978; Lichtenhan et al., 2013). With 100 μM IEM-1460, changes in CAP threshold across frequencies from 1 to 20 kHz were very small (<5 dB SPL; Figure 4B) and within test-retest variability, despite considerable reduction in CAP amplitudes at 2, 8, and 16 kHz (Figures 4C,D). For tones at levels within 5–10 dB of threshold, baseline-normalized CAP amplitude reduction and baseline-normalized CAP latency increase tended to be smaller than for tones at 45–50 dB super-threshold levels (Figure 4D), suggesting that responses to low-level tones may have been inhibited less than responses to high-level tones.

### Reduction of Auditory Nerve Activity With Systemic Delivery of IEM-1460

**Figure 5:**
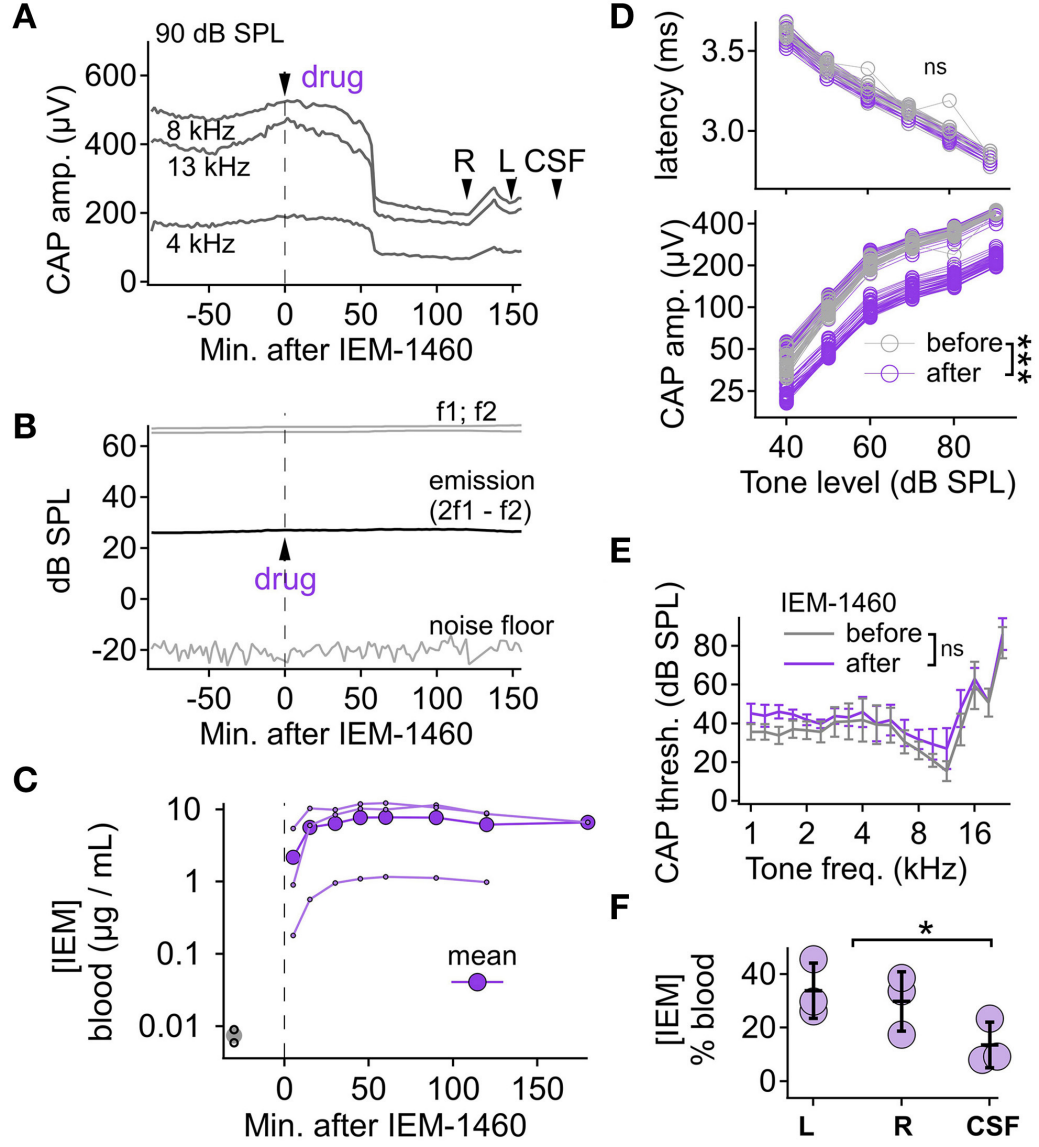
Pharmacokinetic analysis and auditory functional measurements with systemic IEM-1460. **(A)** After baseline recording, IEM-1460 was injected (IP, 13.5 mg/kg) at time zero. CAP amplitude measured every 2 min for 90 dB SPL tones at 4, 8, and 13 kHz. At the end of the experiment, perilymph samples were taken from right (R) and left (L) cochleae before taking the CSF sample at times indicated. CAP N1-P1 amplitude decreased after drug injection. **(B)** Distortion product otoacoustic emissions (2f_1_-f_2_) remained stable after drug injection. **(C)** Blood plasma levels of IEM-1460 increased to a sustained maximum within 30 min of injection (*N* = 3 animals). **(D)** CAP P1 latency was not significantly different (*p* = 0.421, Wilcoxon, *N* = 4) after drug injection (upper) while CAP N1-P1 amplitude was significantly reduced (*p* < 0.001, Wilcoxon, *N* = 4) (lower). **(E)** CAP threshold as a function of cochlear frequency was not significantly different (*p* > 0.100 across all frequency comparisons, Mann-Whitney *U, N* = 4) before and after systemic injection of IEM-1460. **(F)** Perilymph and CSF levels, as % of IEM-1460 concentration in blood, were not significantly different from each other (*p* = 0.670; Friedman test; *N* = 3). *p < 0.05; ***p < 0.001.

Next, we asked if IEM-1460 would produce similar effects when administered systemically (Figure 5). Responses to IP injection of IEM-1460 (13.5 mg/kg) generally consisted of a 20–50 min delay followed by a CAP reduction of 20–50% from baseline within 10–30 min that persisted for up to 2 h at termination of the experiment (e.g., Figure 5A). Blood was sampled throughout the experiment and drug levels were measured using LC/MS. The concentration of IEM-1460 in blood plasma reached a plateau within ~30 min to a level of ~7 μg/mL, on average, that was maintained for the duration of the experiment (Figure 5C). Approximately 2 h after IP injection of IEM-1460, perilymph was collected from the right and then the left cochlea, followed by collection of CSF. LC/MS measured perilymph levels at 17–45% of blood levels, significantly greater than CSF at 8–23% of blood levels (Figure 5F; *p* = 0.029, *N* = 3). Seven μg/mL IEM-1460 corresponds to 23.8 μM in blood, and ~4–11 μM of IEM-1460 in perilymph (see Discussion).

To assess the effect of systemic IEM-1460 on cochlear output (CAP threshold, amplitude, and latency) we compared responses during stable periods before and after IP injection (i.e., before and after the delayed reduction in CAP amplitude induced by the compound). The data were not normally distributed (Kolmogorov-Smirnov test), thus, the Wilcoxon signed-rank test was used to compare CAP amplitudes and latencies, before and after drug injection, in response to tones at different levels for a given frequency. For all frequencies, the CAP amplitude was reduced after drug injection, significantly for each tone level (*p* < 0.001; Wilcoxon with Bonferroni correction for adjusting alpha = 0.05/16, *N* = 4). In the exemplary recording shown in Figure 5D, lower, the mean difference between pre- and post-injection CAP amplitudes ranged 19.1–269 μV across tone levels. The differences scaled with tone level, such that the relative reduction of CAP amplitude was similar (~50%) for probes of 40–90 dB SPL. For CAP latency, there was no statistically significant difference at any tone level (*p* > 0.100 for all tone levels; Wilcoxon with Bonferroni correction, *N* = 4) (Figure 5D, upper). While CAP amplitude was reduced, distortion product otoacoustic emissions (DPOAEs) were unaffected (Figure 5B), suggesting that cochlear mechanics and outer hair cell function were relatively unaffected by IP injection of 13.5 mg/kg IEM-1460. CAP thresholds at each frequency tested from 1 to 22 kHz were not statistically different after IEM-1460 administration (*p* > 0.100 across all frequency comparisons; Mann-Whitney U-test, *N* = 4; Figure 5E). In conclusion, like cochlear perfusion (Figure 4), systemic IEM-1460 (Figure 5) reduced CAP amplitude by as much as 50% with minimal effect on CAP threshold. In contrast to local delivery, where a 10% increase in CAP latency was seen with IEM-1460 perfusion, we did not observe a significant CAP latency shift for systemic dosing.

### Systemic IEM-1925 Is Similarly Potent and Potentially Less Toxic Than IEM-1460

**Figure 6:**
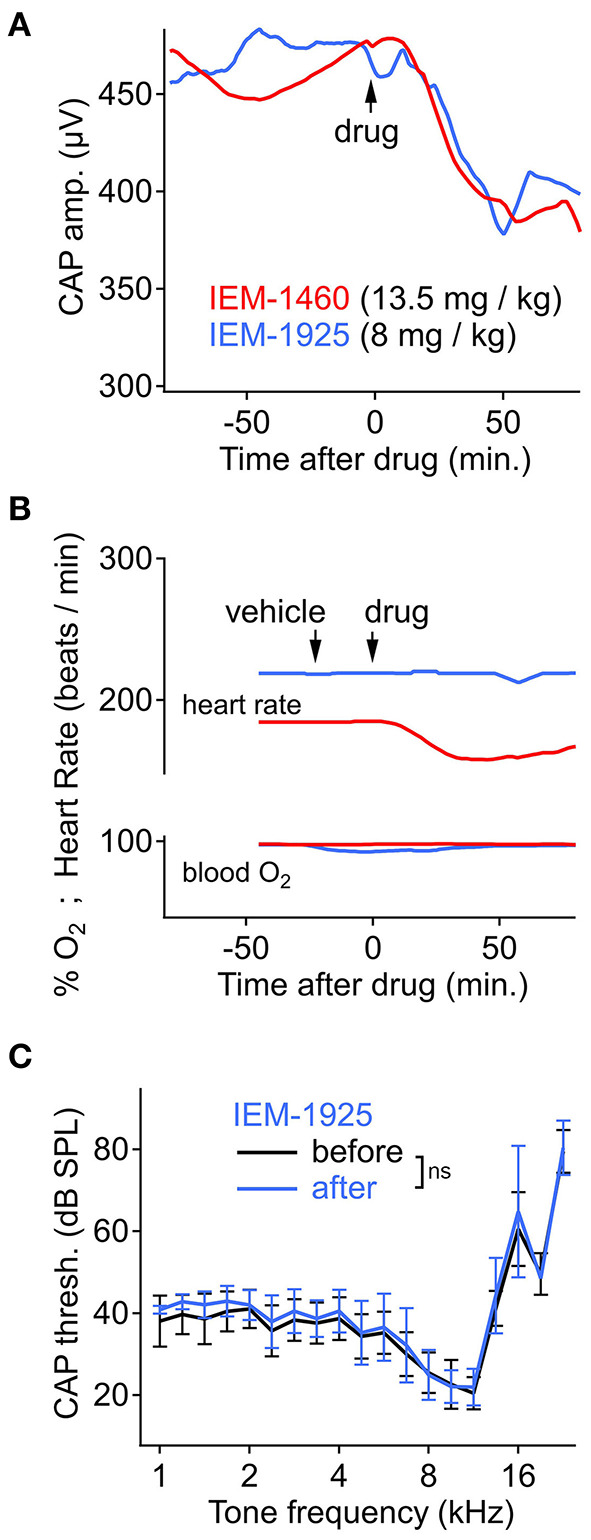
Cochlear and cardiopulmonary physiology during systemic IEM-1460 or IEM-1925. **(A)** CAP amplitude over time, showing significant reduction after intraperitoneal (IP) injection of drug (*p* < 0.001, Wilcoxon, *N* = 4). Stimuli were 8 kHz tone bursts at 90 dB SPL. *N* = 4 animals per compound. **(B)** Heart rate and blood oxygen level before and after IP injection of drug or vehicle control in an exemplary experiment where heart rate was temporarily reduced by 15% (*p* < 0.001, Wilcoxon, *N* = 4) following IP injection of 13.5 mg/kg IEM-1460 (red trace). **(C)** CAP threshold as a function of cochlear frequency was not significantly different after systemic injection of IEM-1925 (*p* > 0.100 across all tested frequencies, Mann-Whitney *U, N* = 4).

The adamantane derivative IEM-1460 is less potent on CP-AMPARs than the phencyclidine derivative IEM-1925, but the two compounds have similar apparent selectivity for CP- over CI-AMPARs (~300-fold) (Tikhonov et al., 2000). We asked if the more potent IEM-1925 could reduce CAP amplitude after systemic injection, like IEM-1460, at a lower dose. Systemic delivery of IEM-1925 (8 mg/kg, IP) reduced CAP amplitude to a similar extent as IEM-1460 (13.5 mg/kg, IP). On average, CAP amplitude reduction began within 10–15 min and was reduced by ~ 20% after ~1 h (Figure 6A, *N* = 4 animals per compound). The CAP amplitude after injection of IEM-1925, similarly to IEM-1460, was significantly reduced after drug injection for all tone levels and frequencies (*p* < 0.001 across all tone levels, Wilcoxon signed-rank test with Bonferroni correction for adjusting alpha = 0.05/3, *N* = 4). Blood oxygenation and heart rate were monitored along with cochlear physiology during the time course of the experiment. Changes in blood oxygenation were <5% and not synchronized to drug injection (*p* = 0.562, Wilcoxon signed-rank test, *N* = 4). However, IEM-1460 produced a temporary, significant reduction in heart rate (Figure 6B) by as much as 15% (*p* < 0.001, Wilcoxon signed-rank test, *N* = 4). No such reduction in heart rate was observed for IEM-1925. Like IEM-1460, systemic injection of IEM-1925 had minimal effect on CAP thresholds (*p* > 0.100 across all frequency comparisons, Mann-Whitney *U*-test, *N* = 4; Figure 6C).

We tested the metabolic stability of the compounds with *in vitro* liver microsome stability assays (Table 1, right). In mouse, guinea pig, and human liver microsomes, IEM-1460 and IEM-1925 were very stable, exhibiting half-lives of >300 min. We evaluated the compounds for potential toxicity with assays that test for inhibition of off-target ion channels and receptors. With radioligand binding assays, we screened for activity of the test compounds IEM-1460 and IEM-1925 on a set of ion channels and receptors to be avoided in the heart and central nervous system (Table 1, left). Ion channels and ionotropic receptors included voltage gated K_V_11.1 (delayed rectifier K^+^ channel in cardiomyocytes), the ionotropic NMDAR and AMPAR, and the nicotinic acetylcholine receptors (AChR) alpha 4/2 and alpha 7. Metabotropic receptors included adenosine, histamine, and metabotropic AChRs. At a concentration of 1 μM, IEM-1460 inhibited radioligand binding to the M4 and M3 AChRs by 37 and 15%, respectively. All other off-target interactions were inhibited by <5%. For IEM-1925 at 1 μM, also, the M4 AChR was the biggest hit at 19% inhibition. Inhibition of AMPA binding to AMPARs by IEM-1925 was 9.5%, while all other competitive binding interactions were inhibited by <5% (Table 1). This panel of off-target binding assays suggests that IEM-1925 may have an enhanced safety profile relative to IEM-1460.

**Table 1:** *In vitro* off-target binding assays and metabolic stability of IEM-1460 and IEM-1925.

**Radioligand binding assays**	**Antagonist: IEM-1925 (1 μM)**	**Antagonist: IEM-1460 (1 μM)**	**Liver microsome stability assays**	**Half-life (min)**
Receptor/Channel	Mean% inhibition (*N =* 2)	Mean% inhibition (*N =* 2)		Mean (*N =* 2)
hERG potassium (K_v_ 11.1), human	<1	<5		
NMDA glutamate, rat	<1	<1	Human	
AMPA glutamate (GluA2-containing), rat	9.5	<1	IEM-1460 IEM-1925	>300 >300
A1 adenosine GPCR, human	<1	<1		
H1 histamine GPCR, human	<1	<1	Guinea pig	
H2 histamine GPCR, human	<1	<1	IEM-1460 IEM-1925	>300 >300
H3 histamine GPCR, human	<5	<1		
H4 histamine GPCR, human	<5	<1		
M1 acetylcholine GPCR, human	<1	<1	Mouse	
M2 acetylcholine GPCR, human	<1	<1	IEM-1460 IEM-1925	>300 >300
M3 acetylcholine GPCR, human	<5	15		
M4 acetylcholine GPCR, human	19	37		
M5 acetylcholine GPCR, human	<5	5.2		
nAChR alpha4/beta2, human	<1	<1		
nAChR alpha7, human	<2	<1		

*Left: Radioligand binding assays show% inhibition of binding of the receptor-specific radioligand (see Methods) to the off-target ion channel or receptor by 1 μM IEM-1460 or IEM-1925. Right: Stability of 1 μM IEM-1460 or IEM-1925 in the presence of mouse, guinea pig, or human liver microsomes at 37°C*.

## Discussion

### Antagonizing CP-AMPARs to Prevent Glutamate Excitotoxicity?

Ischemia, noise exposure, or perfusion of the guinea pig cochlea with glutamate, kainate, or the specific agonist AMPA produces osmotic swelling of ANF postsynaptic terminals in the organ of Corti underneath IHCs (Puel et al., 1994, 1998; Figure 3). Landmark studies used electron microscopy after perfusion of glutamate receptor agonists and antagonists into the basal cochlear turn. These chemical lesion experiments showed that ANF terminal swelling was prevented by pre-perfusion with DNQX, a pan-AMPAR antagonist, but not with the N-methyl-D-aspartate receptor (NMDAR) antagonist D-AP5, demonstrating that glutamatergic excitotoxicity is mediated via AMPARs (Puel et al., 1994). AMPAR agonists bring positive ions into the ANF terminal and dendrite. The resulting depolarization of the nearby heminode triggers additional Na^+^ influx into the thin fiber through voltage-gated channels. The spike-generating heminodes are located at the habenula perforata (HP), just before the myelin begins, where ANFs enter the osseous spiral lamina (OSL; Figures 1B,C). These myelinated peripheral axons propagate spikes toward bipolar cell bodies in the spiral ganglion. Unlike a conventional neuron, the spike-generating heminode of the ANF is separated from the excitatory ribbon synapse not by a cell body but instead by only a ~20 μm length of dendrite with submicron diameter (Figures 1B,C). The tight anatomical and electrical coupling between the synapse and spike generator, as well as the alignment of heminodes at the HP where the myelin begins, is important for synchronous activation of ANFs in response to sound (Rutherford et al., 2012; Kim and Rutherford, 2016; Wan and Corfas, 2017). This may also render the ANF dendrite vulnerable during high rates of activity, when influx of positive ions at the synapse and heminode leads to osmotic terminal swelling and synapse disintegration.

AMPARs mediate most of the fast excitatory chemical transmission in the brain. As GluA2 is the 1st or 2nd most abundant subunit, the great majority of AMPAR tetramers are thought to include at least one GluA2 subunit, >99% of which are Q/R edited at a critical residue in the pore's selectivity filter to reduce overall channel conductance and relative Ca^2+^ permeability (Geiger et al., 1995; Schwenk et al., 2012; Shanks et al., 2012; Wright and Vissel, 2012). Thus, AMPARs are generally of the relatively low-conductance variety and impermeable to Ca^2+^ due to inclusion of edited GluA2 in the tetramer. An exception to this general rule are some neurons in the ascending auditory pathway, where synapses with fast kinetics of transmission support rapid and precise signaling of auditory information using AMPARs that appear to rely mainly on GluA3 and GluA4 (Otis et al., 1995; Yang et al., 2011; Rubio et al., 2017; Lujan et al., 2019). Absence of GluA2 in the tetramer results in an AMPAR with larger currents, faster kinetics, and greater permeability to Ca^2+^. While some synaptic mechanisms appear to utilize CP-AMPARs, aberrant expression of CP-AMPARs has been implicated in many neurological disorders where excessive activity leads to neurodegeneration (Weiss, 2011), including but not limited to epilepsy (Grooms et al., 2000; Rajasekaran et al., 2012; Malkin et al., 2016), ischemia (Kwak and Weiss, 2006), traumatic brain injury (Spaethling et al., 2008), and illicit substance addiction and withdrawal (Pistillo et al., 2016; Wolf, 2016). The *in vitro* binding assays (Table 1) suggest that CNS toxicity of IEM-1925 may be limited based on weak interference of binding of CNS receptors to their natural ligands. However, subsequent studies of IEM-1925 or its derivatives must evaluate binding to the pore of off-target channels. It will be imperative to thoroughly evaluate CNS toxicity of any drug candidates with behavioral studies as well.

The fish lateral-line organ and the mammalian organ of Corti are examples of peripheral sensory organs that mediate fast acoustic signals via synapses between sensory hair cells and primary afferent neurons which appear to utilize GluA2-lacking, CP-AMPARs (Sebe et al., 2017; Hu et al., 2020). The faster, larger currents of CP-AMPARs are expected to drive the postsynaptic neuron to spike threshold more precisely with respect to temporal changes in the hair cell receptor potential, which may be important for mechanisms of sensory encoding such as sound source localization in the horizontal plane mediated by phase-locking (Goutman, 2012; Rutherford et al., 2012, 2021; Li et al., 2014). In fish and mammals, excessive activation of AMPARs can lead to postsynaptic terminal swelling and synaptic disintegration, suggesting that CP-AMPARs may be targeted for prevention of glutamatergic excitotoxic damage. Indeed, local delivery of the CP-AMPAR antagonist IEM-1460 prevented damage induced by chemical lesion or acoustic trauma (Sebe et al., 2017; Hu et al., 2020). However, the selectivity of IEM-1460 for CP-AMPARs is concentration dependent (Tikhonov et al., 2000). At high concentration it will block all AMPARs thus preventing all auditory nerve activity and hearing itself, as demonstrated by perfusion of different drug concentrations into scala tympani (Figure 4A). For prevention of damage to hearing, local delivery of therapeutic agents to the inner ear is likely too invasive. Therefore, here we asked if systemic administration of CP-AMPAR antagonists could safely reduce afferent synaptic activity in the inner ear without elevating auditory thresholds. We further asked if the level of drug in perilymph was consistent with selective blockade of CP-AMPARs.

### Reduction of Auditory Nerve Activity Without Elevation of Hearing Threshold

We found that systemic administration of the CP-AMPAR antagonists IEM-1460 or IEM-1925 reduced CAP amplitude (Figures 5A,D, 6A) without significantly affecting auditory thresholds (CAP audiogram, Figures 5E, 6C) or otoacoustic emissions (i.e., DPOAEs, Figure 5B). Local or systemic dosing of IEM-1460 attenuated CAP amplitudes in response to low-level and high-level tones (Figure 4D). In contrast, the medial olivocochlear (MOC) efferent reflex inhibits the CAP primarily in response to low-level sounds and is thus thought to provide only limited protection against acoustic trauma (Puria et al., 1996; Guinan, 2006; Lichtenhan et al., 2016b). While middle ear muscle reflexes are activated at high sound levels and protectively attenuate sound transmission through the middle ear, these muscles fatigue over prolonged exposure and have a long latency (i.e., 10–15 ms) that does not seem to protect against sudden loud sounds or prolonged exposure (Simmons, 1960; Ryan et al., 1994). Attenuation of the CAP at high sound levels, as seen here with antagonism of CP-AMPARs, could be protective when the MOC or middle ear reflexes are ineffective.

In animal models, noise exposure can permanently change cochlear physiology in part through effects on synapses that don't necessarily cause an immediate permanent threshold shift but do reduce CAP or wave-I amplitude. Such effects of noise which may be compounded by aging have been termed “hidden hearing loss” because they aren't detected by pure-tone audiometry (Liberman, 2020). Data from audiometrically normal adults shows that people with smaller auditory nerve or subcortical responses perform relatively poorly on hearing speech in the presence of background noise (Bharadwaj et al., 2015; Bramhall et al., 2015; Stamper and Johnson, 2015; Grant et al., 2020). However, this is still controversial as many studies in humans have failed to find significant relationships between speech perception and the amplitude of the auditory nerve action potential (Prendergast et al., 2017; Guest et al., 2018; for review see Bramhall et al., 2019). An outstanding question is if pharmacologic reduction of auditory nerve activity, without threshold elevation, reduces intelligibility of speech and other sounds. If not, then it may be possible to protect hearing by dampening activity without compromising hearing function. If sound intelligibility is reduced during protection, the benefits of protection may outweigh the disadvantage of temporarily reduced audibility. Subsequent studies should evaluate the time course of reduced auditory nerve activity after systemic administration, as the current studies were terminated before the effect wore off.

### Relationship Between CP-AMPARs and ANF Physiology

Each cochlear afferent synapse has a relatively large PSD, parts of which appear to lack GluA2 based on subunit-specific pharmacology and microscopy (Sebe et al., 2017; Hu et al., 2020). In mouse, after ~2.5 days of sustained intracochlear delivery of IEM-1460, Hu et al. (2020) found prevention of synapse loss following exposure to synaptopathic noise. Interestingly, IEM-1460 prevented synapse loss and loss of wave-I amplitude without significantly reducing wave-I amplitude *during* the protection. This suggested that GluA2-containing AMPARs can mediate hearing function without reduction of auditory nerve activity alongside chronic, protective antagonism of GluA2-lacking CP-AMPARs. Here, acute local or systemic drug delivery reduced sound-evoked activity (i.e., the CAP amplitude) when IEM-1460 was delivered at concentrations selective for CP-AMPARs (Figures 4–6). This is somewhat surprising, given the lack of ABR wave-I reduction during protection in Hu et al. (2020).

To explain the difference between the present findings and those in Hu et al. (2020), specifically the effect of IEM-1460 on suprathreshold hearing, one possibility is a species difference. If the guinea pig cochlea has more CP-AMPARs, and thus more block of transmission by CP-AMPAR antagonists, then IEM-1460 may reduce ANF activity in the guinea pig and not the mouse. A 2nd possibility is the effect of chronic vs. acute drug delivery. If synaptic adaptation compensated during chronic dosing by inserting more GluA2-containing, CI-AMPARs that are insensitive to IEM-1460, then ABR wave-I (or CAP) amplitudes may have been maintained in the continuous presence of the drug. A 3rd possibility is a difference in the concentration of compound between experiments. In Hu et al. (2020) the concentration of drug in perilymph was unknown as a minipump was used to elute the compound into scala tympani. In the present study, drug concentration was controlled by local perfusion of known concentrations into scala tympani (Figure 4) or by measuring concentrations in cochlear fluid samples (Figure 5). If the concentration was considerably lower in the minipump experiment, this could explain the lack of wave-I reduction. We find this possibility unlikely because a well-established simulation model of drug concentration in scala tympani of the mouse suggested a mean concentration of ~ 58 μM (https://alecsalt.com/index.php/simulations/fluidsim4). A fourth consideration is different anesthesia. Here we used isoflurane, whereas Hu et al. (2020) used ketamine/xylazine.

A separate question is: how were the mouse synapses protected in the absence of reduced ABR wave-I during chronic presence of the drug (Hu et al., 2020)? Subunit adaptation may have resulted in fewer CP-AMPARs, in which case less Ca^2+^ entry relative to Na^+^ entry could reduce noise-induced excitotoxicity. As well, if the ANF EPSC is much larger than required to reach spike threshold, as suggested by *ex vivo* electrophysiology (Rutherford et al., 2012), then the excitotoxic EPSC may be safely reduced in level without inhibiting action potential generation. In this scenario, the number of active ANFs would not be reduced, but the smaller EPSC may increase spike latency. Interestingly, CAP latency was slightly increased with local delivery of IEM-1460 in guinea pig (Figure 4D), although not significantly for systemic delivery (Figure 5D).

### A Mixture of Ca^2+^-Permeable and Ca^2+^-Impermeable AMPARs at the Hair Cell Afferent Synapse

Tight spatial coupling between the ribbon synapse and spike-generating heminode (Figure 1), along with small fiber diameter and high rates of activity, results in ANF dendritic vulnerability to osmotic imbalance that may be exacerbated by the presence of CP-AMPARs. The larger Na^+^ and Ca^2+^ currents through CP-AMPARs, while presumably important for fast signaling, also place greater metabolic demands on the ANF through energy consumption by the pumps that maintain ionic gradients. The Na^+^/K^+^-ATPase on the ANF plasma membrane (labeling ANFs in Figures 1C, 3, abbreviated NKA) uses energy to pump Na^+^ out of the cytoplasm (Chow and Forte, 1995; Blanco and Mercer, 1998). Calcium is particularly expensive to handle. The Na^+^/Ca^2+^ exchanger is an antiporter membrane protein that removes Ca^2+^ from cells by allowing Na^+^ to flow into cells. A single Ca^2+^ ion is exported for the import of three Na^+^ ions (Fettiplace and Nam, 2019) which must then be pumped out by the Na^+^/K^+^-ATPase. We suggest this is one way that excessive activity of CP-AMPARs produces oxidative stress through energy consumption.

In rat hippocampus, on non-pyramidal cells (expressing mainly GluA2-lacking CP-AMPARs) the IC_50_ for IEM-1460 and IEM-1925 is ~3 and ~1 μM, respectively, while on pyramidal cells (expressing mainly GluA2-containing CI-AMPARs) the IC_50_ is > 500 and > 200 μM, respectively (Tikhonov et al., 2000). Here, in guinea pig, with CAP measurements during measured or defined concentrations of the compounds in perilymph, we found reduction of ANF activity at concentrations consistent with the presence of a mixture of CP- and CI-AMPARs. Figure 5C shows that IEM-1460 was sustained in blood at ~7 μg/mL, on average after IP injection. Figure 5F shows that perilymph levels were ~30% of blood levels. IEM-1460 at 2 μg/mL is ~7 μM, near to the IC_50_ for CP-AMPARs but far from the IC_50_ for CI-AMPARs. This level of systemic dosing reduced the CAP by ~20%, on average, consistent with the presence of CP-AMPARs. In other experiments, cochlear perfusion of nominally 100 μM IEM-1460 (Figure 4) reduced CAP amplitude by ~55%. This concentration is still well below the IC_50_ for CI-AMPARs (>500 μM), and well above the IC_50_ for CP-AMPARs (~3 μM). Taken together, we conclude that afferent synapses in the guinea pig cochlea contain a mixture of CP- and CI-AMPARs, as in the mouse (Hu et al., 2020). It will be important to test this directly with patch-clamp recordings of EPSCs from ANF postsynaptic terminals in the cochlea, in subsequent studies.

### Physicochemical Properties of IEM-1460 and IEM-1925 for Oral Bioavailability and Inner Ear Permeation

There are currently no FDA-approved drugs for prevention of noise-induced hearing loss or other noise-induced hearing disorders. A limited understanding of unique targets within the inner ear hinders drug design for systemic delivery, thus much of current focus has been on local delivery (McCall et al., 2010; Nyberg et al., 2019). Unfortunately, intracochlear delivery (i.e., directly to the inner ear) is too invasive for chronic or sub-chronic dosing for prevention of damage to healthy ears. Middle ear delivery may introduce drugs into the cochlea if they permeate the round window into scala tympani, however, infiltration of CSF into perilymph at the base of the cochlea near the round window prevents drugs from making it very far up the cochlea resulting in a gradient from base to apex in guinea pig that effectively limits drug delivery to the base. When drugs enter the cochlea through blood, there is no such gradient. For systemic dosing, a favorable inner ear drug candidate would target a specific receptor within the inner ear with limited CNS expression, while reaching the inner ear at efficacious concentrations.

**Figure 7:**
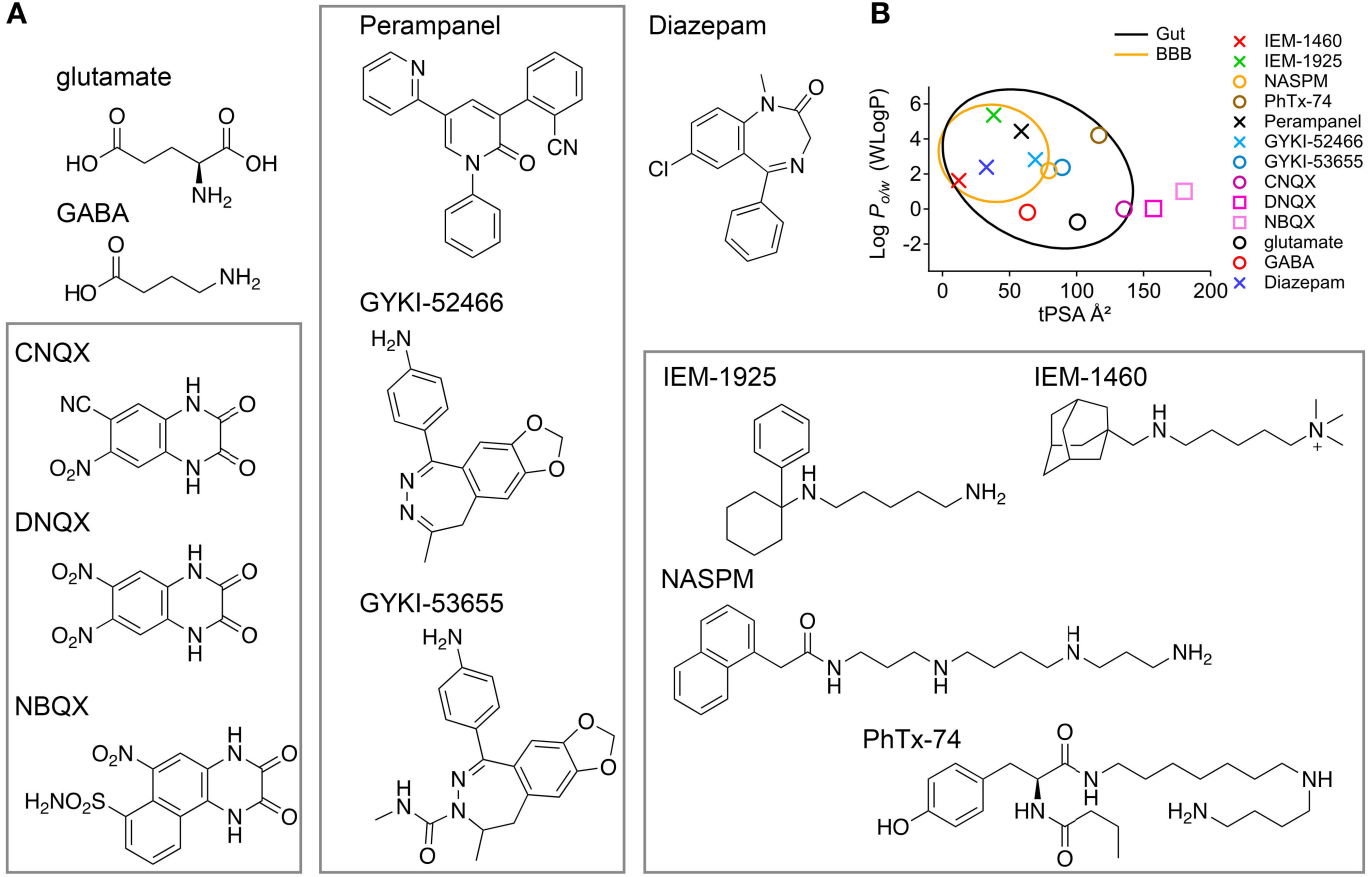
Molecular structures and CNS accessibilities of AMPAR antagonists (rectangles). **(A)** The agonists glutamate and GABA are small but not very lipophilic. CNQX, DNQX, and NBQX are competitive AMPAR antagonists likely too polar to pass the gut. Perampanel, GYKI-52466, and GYKI-53655 are non-competitive and non-selective AMPAR antagonists that function as negative allosteric modulators. IEM-1460, IEM-1925, NASPM, and PhTx-74 are non-competitive and selective CP-AMPAR antagonists that function as open-channel pore blockers. **(B)** The so-called “boiled egg plot” uses a hydrophilicity metric developed by Wildman and Crippen (Y-axis, WLogP) and topological polar surface area (X-axis, tPSA) to predict gastrointestinal absorption and brain penetration. Egg-white region: high probability of absorption by the gastrointestinal tract; Egg-yolk region: highest probability of permeation through the blood-brain barrier (BBB). The drugs Perampanel and Diazepam are well within the egg yolk. Glutamate and GABA, although made in the brain, are predicted not to penetrate the BBB. IEM-1460 and IEM-1925 are less potent than NASPM and PhTx-74 (μM vs. nM potency) but more likely to permeate the CNS.

The data in this study show that IEM-1460 and IEM-1925 may have favorable pharmacokinetic properties for targeting CP-AMPARs in the inner ear with systemic dosing (Figures 5C,F), and that IEM-1925 may be less toxic than IEM-1460 (Figure 6B; Table 1). Based on their computed hydrophilicity (CLogP or WLogP, Wildman and Crippen, 1999) and polarity (tPSA), IEM-1460 and IEM-1925 are predicted to be absorbed by the gastrointestinal tract and cross the blood brain barrier (BBB) (Figure 7). In Figure 7B, the egg-white region is the physicochemical space of molecules with highest probability of being absorbed by the gastrointestinal tract, and the egg-yolk region is the physicochemical space of molecules with highest probability to further permeate into the brain (Zoete et al., 2016; Daina et al., 2017). Like the non-selective AMPAR antagonists Perampanel and GYKI-52466, the selective IEM-1460 and IEM-1925 are in the egg-yolk (i.e., predicted to cross the BBB). These compounds are non-competitive AMPAR antagonists, meaning they don't compete for glutamate's binding site on the ligand-binding domains of other glutamate receptors. Instead, they inhibit by blocking the ion channel pore. Unlike Perampanel and GYKI-52466, IEM-1460 and IEM-1925 are selective for GluA2-lacking AMPARs, restricting their activity to CP-AMPARs. In addition, IEM-1460 and IEM-1925 are open-channel blockers, meaning the channel must open before it can be accessed by the antagonists. Non-competitive, open-channel antagonists block slowly such that the initial component of the response may be weakly affected. They require the channel to spend time in the open conformation for blockade to develop, and thus they should be more effective at blocking AMPAR channels that are stimulated at high frequency. By selectively targeting the *most frequently active* GluA2-lacking AMPARs, it may be possible to inhibit excitotoxicity more so than normal synapse function.

The mechanism by which compounds enter perilymph is not clear. Compounds that penetrate the BBB can potentially enter perilymph via the cochlear aqueduct, neuronal pathways, or lymphatic pathways (Salt and Hirose, 2018). The blood-labyrinth barrier (BLB) regulates the chemical composition of endolymph and perilymph via the stria vascularis and spiral ligament (Juhn and Rybak, 1981; Suzuki et al., 2002). It has been hypothesized through experiments in guinea pigs that noise exposure may increase macromolecular transport in the stria vascularis such that systemically administered macromolecules can be more readily transported into the inner ear by noise exposure (Suzuki et al., 2002). This phenomenon can potentially be used for drug design and targeting. The BLB may be leakier than the BBB, in general (Hirose et al., 2014; Wu et al., 2014). After systemic injection of IEM-1460, perilymph levels trended somewhat higher than CSF levels (Figure 5F). Nonetheless, for an oral drug the challenge will be to find compounds without significant CNS side effects. Although IEM-1925 (8 mg/kg) produced similar CAP reduction as IEM-1460 (13.5 mg/kg), LC/MS did not detect significant IEM-1925 in perilymph or CSF. Very low levels of IEM-1925 were detected in blood, despite successful calibration of concentrations in artificial perilymph and blood plasma (Figure 2), suggesting the IEM-1925 may have been metabolized, bound to tissue, or otherwise masked *in vivo*. Both compounds had very long half-lives in liver microsome stability assays (Table 1), suggesting IEM-1925 metabolism in the guinea pig that was not accounted for in liver microsomes. Future studies should investigate candidate metabolites with activity at CP-AMPARs. Analysis of our PK data from Figure 5C estimates an elimination half-life of 118 min for IEM-1460 in blood. Although levels of IEM-1925 in blood were low, we were still able to estimate a half-life of 68 min. Subsequent studies will measure the recovery of CAP amplitudes in the hours after drug administration.

In addition to CLogP and tPSA, other molecular properties important for a drug's pharmacokinetics in the human body including their absorption, distribution, metabolism, and excretion (ADME) are molecular weight (MW), acidity (pKa), number of hydrogen bond donors (HB-donor) and acceptors (HB-acceptor), and the number of rotatable bonds (Table 2). Lipinski's rule of five, also called Pfizer's rule of five, is a rule of thumb to determine how likely a compound is to be an orally active drug in humans (Lipinski et al., 2001). The rule states that there should be no more than five hydrogen bond donors, no more than 10 hydrogen bond acceptors, a MW <500 Daltons, and CLogP <5. Table 2 shows that IEM-1460 and IEM-1925 have good physicochemical properties for drug-likeness.

**Table 2:** Physicochemical properties for drug-likeness.

**Name**	**Computed property**						
	**MW**	**CLogP**	**pKa**	**HB-donor**	**HB-acceptor**	**Polar surface area**	**Rotatable bonds**
Lipinski (oral)	<500	≤5	3–10	≤5	≤10	≤140	≤14
CNS bioavailability	≤400	≤4	7.5–10	≤3	≤7	≤60	≤8
PhTX-74	449	3.3	9.8	7	6	116	19
IEM-1460	294	3.7	10.5	1	2	12	8
IEM-1925	260	3.7	10.5	3	2	38	7

*Physicochemical properties were computed for IEM-1460, IEM-1925, and philanthotoxin 74 (PhTX-74). They are compared with the Lipinski rules of thumb for oral absorption and CNS bioavailability*.

## Data Availability Statement

The raw data supporting the conclusions of this article will be made available by the authors, without undue reservation.

## Ethics Statement

The animal study was reviewed and approved by Institutional Animal Care and Use Committee at Washington University in St. Louis (20180133, 20190035).

## Author Contributions

MR: study concept and organization. JL, AS, SG, RD, CL, JH, AW, and MR: research design. JL and MR: funding acquisition. CL, JH, AW, MR, JL, and AS: experimental data collection. AW, CL, SG, RD, AS, JL, and MR: data analysis and tools. MR and AW: manuscript inception. AW, JL, CL, RD, AS, and MR: draft revision. All authors contributed to the article and approved the submitted version.

## Conflict of Interest

A provisional patent application titled “Targeting Calcium-Permeable AMPA Receptors for Inner Ear Therapy with IEM-1460 and Related Compounds” was filed on 23 December 2019. Some data presented in this paper was cited in the application.
